# Sensorimotor Rehabilitation Promotes Vestibular Compensation in a Rodent Model of Acute Peripheral Vestibulopathy by Promoting Microgliogenesis in the Deafferented Vestibular Nuclei

**DOI:** 10.3390/cells10123377

**Published:** 2021-12-01

**Authors:** Emna Marouane, Nada El Mahmoudi, Guillaume Rastoldo, David Péricat, Isabelle Watabe, Agnès Lapôtre, Alain Tonetto, Frédéric Xavier, Olivier Dumas, Christian Chabbert, Vincent Artzner, Brahim Tighilet

**Affiliations:** 1 Aix-Marseille Université-CNRS, Laboratoire de Neurosciences Cognitives, LNC UMR 7291, Centre Saint-Charles Case C, 3 Place Victor Hugo, CEDEX 03, 13331 Marseille, France; emna.marouane@gmail.com (E.M.); nada.EL-MAHMOUDI@univ-amu.fr (N.E.M.); guillaume.RASTOLDO@univ-amu.fr (G.R.); isabelle.watabe@univ-amu.fr (I.W.); agnes.lapotre@univ-amu.fr (A.L.); frederic.xavier@etu.univ-amu.fr (F.X.); christian.CHABBERT@univ-amu.fr (C.C.); 2 BIOSEB ALLCAT Instruments, Couperigne, 13127 Vitrolles, France; vartzner@bioseb.com; 3 Institute of Pharmacology and Structural Biology (IPBS), University of Toulouse, CNRS, 31400 Toulouse, France; david.pericat@ipbs.fr; 4 Fédération de Recherche Sciences Chimiques Marseille FR 1739, Pôle 18 PRATIM, CEDEX 03, 13331 Marseille, France; alain.tonetto@univ-amu.fr; 5 GDR Physiopathologie Vestibulaire—Unité GDR2074, CNRS, 13003 Marseille, France; ol.dumas@wanadoo.fr

**Keywords:** vestibular rehabilitation, vestibular compensation, posturolocomotor instability, cell differentiation, microglial reaction, neurogenesis, vestibular nuclei

## Abstract

Acute peripheral vestibulopathy leads to a cascade of symptoms involving balance and gait disorders that are particularly disabling for vestibular patients. Vestibular rehabilitation protocols have proven to be effective in improving vestibular compensation in clinical practice. Yet, the underlying neurobiological correlates remain unknown. The aim of this study was to highlight the behavioural and cellular consequences of a vestibular rehabilitation protocol adapted to a rat model of unilateral vestibular neurectomy. We developed a progressive sensory-motor rehabilitation task, and the behavioural consequences were quantified using a weight-distribution device. This analysis method provides a precise and ecological analysis of posturolocomotor vestibular deficits. At the cellular level, we focused on the analysis of plasticity mechanisms expressed in the vestibular nuclei. The results obtained show that vestibular rehabilitation induces a faster recovery of posturolocomotor deficits during vestibular compensation associated with a decrease in neurogenesis and an increase in microgliogenesis in the deafferented medial vestibular nucleus. This study reveals for the first time a part of the underlying adaptative neuroplasticity mechanisms of vestibular rehabilitation. These original data incite further investigation of the impact of rehabilitation on animal models of vestibulopathy. This new line of research should improve the management of vestibular patients.

## 1. Introduction

At rest and during displacements, postural adaptations must be made in real time to manage one’s balance in the face of environmental disturbances. To prevent falling in front of possible obstacles, a multi-sensory integration is ensured, involving visual, vestibular, and proprioceptive systems. The vestibular nuclei (VN) located in the brain stem receive afferences from all these sensory modalities. Integration of these information by the VN allows to provide posturolocomotor responses adapted to the external environment [1]. Because of this crucial integrative role, unilateral impairment of the vestibular system leads to a vestibular syndrome that is particularly disabling for patients’ daily life and which is characterized by stereotypical symptoms [2]. Sudden alteration of the sensory inputs arising from peripheral vestibular receptors causes, among other things, postural asymmetry and several locomotor deficits [3,4,5]. It is now well admitted that acute vestibular syndrome originates from electrophysiological asymmetry between intact and deafferented VN and that recovery from this syndrome occurs through a rebalancing of this electrical activity [6,7]. The return to electrophysiological homeostasis, considered the key parameter for vestibular functional recovery, takes place via a mosaic of neuroplasticity mechanisms occurring specifically within the deafferented VN [8]. Among these mechanisms, reactive neurogliogenesis has been observed in numerous studies in rat and feline unilateral vestibular neurectomy (UVN) models [9,10,11,12,13] and favour vestibular function recovery [13]. A peak of cell proliferation occurs in a critical time window, accompanied by an inflammatory response [14], release of neurotrophic factors [11], and increased glial response [9] in the VN. However, despite the expression of these intrinsic mechanisms contributing to the restoration of balance, certain disorders persist over time despite the offered pharmacological therapies offered. [15,16,17]. Nevertheless, numerous clinical studies show the beneficial effects of vestibular rehabilitation protocols, which aim to accelerate vestibular compensation through numerous gait-, balance-, and gaze-stabilization exercises [18,19,20,21,22]. On the other hand, little is known about the cellular mechanisms induced by vestibular rehabilitation protocols in the VN. Vestibular rehabilitation is mainly studied in clinical research, and very few studies are performed in animal models of vestibular pathology. The few existing studies have revealed a decrease in anxiety following balance training in a mouse model of vestibular disorders [23] and an increase in the number of vestibular synapses projecting on the motor neurons of the limb muscles in elderly mice subject to postural imbalance [24]. The effects of rehabilitation protocols in the study of vestibular compensation in rodents has never been studied. In addition, numerous studies highlighted the beneficial effects of physical exercise on neurogenesis and brain plasticity in numerous models of brain pathologies [25,26,27].

This study was designed with the aim to investigate the impact of a sensorimotor rehabilitation protocol adapted to a UVN rodent model on the posturolocomotor recovery during vestibular compensation and the provoked cellular mechanisms in the deafferented medial vestibular nuclei (MVN).

## 2. Materials and Methods

### 2.1. Animals

The experiments were performed on 25 Long Evans male rats of 10–12 weeks old (250/300 g) originating from our own breeding, from parents arising from Charles River (St Germain sur l’Arbresle, France). All experiments were performed in accordance the European directive (2010/63/CE), which sets the regulations for the protection of animals used for scientific purposes, and under the veterinary and the National Ethical Committee supervision (French Agriculture Ministry Authorization: B13-055-25). Every attempt was made to minimize both the number and the suffering of animals used in this experiment. The animals were housed in a large, confined space with 12-h diurnal light variations with free access to water and food. They were housed at the Fédération 3C (Centre Saint-Charles, Aix-Marseille University) animal facility.

The 25 animals were divided into three groups: SHAM (*n* = 7), UVN (*n* = 9), and rehabilitation (*n* = 7) groups. Two animals were excluded from the rehabilitation group because they did not learn to run on the treadmill (one remained motionless, and the other found a way out of the device). See below for the details of procedures and surgery.

### 2.2. Study Design

The behavioural investigations were carried out in two parts: a first quantitative evaluation of the vestibular syndrome with the DWB2^®^ according to [28] and a second qualitative evaluation of the syndrome according to [29]. The rats were manipulated during one week before the first preoperative session. During this session, a quantitative analysis of the postural parameters (reference values) was performed with the DWB2^®^. All the rats then underwent surgery. The behaviour of the three groups was assessed by these two tests throughout the duration of the vestibular syndrome (daily in the critical period from D1 to D3, and then at post-injury days 7, 10, 14, 17, 21, and 30 of the compensated stage).

The rehabilitation group underwent a sensorimotor rehabilitation protocol twice a day from D1 to D10 detailed below. Training consisted of progressive learning to walk on a treadmill on which two obstacle bars were placed sufficiently far apart to allow the animal to execute a complete locomotor cycle. In developing the protocol, we applied the clinical recommendations of a previous study in humans [30], with the aim of optimizing the functional recovery of the rehabilitation group. These recommendations are divided into several points based on the expertise of the authors in the field of vestibular compensation and are followed in our study. Training was performed during the first 10 days post-operative, which covers the entire critical period (D1 to D3) and the beginning of the compensated period in this rodent model [29,30,31,32]. During the critical period, processes of neurogenesis and adaptive plasticity tending towards functional restoration are intensely expressed in the VN [33]. This training goes beyond the critical period to maintain previously observed adaptive processes. The protocol is based on an active task to generate a sensory-motor effort while being adapted to the evolution of vestibular disorders through a gradual increase in the speed of the treadmill. The postural adaptations necessary for the evolution of the rehabilitation task require the integration of multisensory information through the VN. Finally, this training is ecological. Walking with obstacle avoidance reproduces ecological conditions. In this study, we were also interested in the cellular phenotype of the VN resulting from the rehabilitation protocol. The rehabilitation group and the UVN group received a BrdU injection (Bromodeoxyuridine, 200 mg/kg, i.p.) on day 3 post-UVN and were killed at day 30 post-lesion to study vestibular compensation with the survival and the differentiation of the proliferating cells. The BrdU is a structural analogue of thymidine that is incorporated into the DNA of newly synthesised cells. It is a marker of cell proliferation.

### 2.3. Surgery

Left UVN was performed on 18 rats following the surgical procedure previously reported in the literature [9,28,29,31,32,34]. A tympanic bulla approach gave access to the vestibular nerve, which was sectioned at its entry into the brainstem. In another 7 sham rats, surgery was stopped at the opening of the tympanic bulla.

### 2.4. Sensory-Motor Rehabilitation

The animals were trained from D1 to D10 twice a day to an obstacle-avoidance task on a treadmill (Columbus Instruments, Colombus, OH, USA, Model Simplex II). The treadmill was modified to add two obstacle bars glued to the floor (dimensions: height = 1.5 cm, width = 13 cm, thickness = 0.7 cm) and separated by 45 cm. The purpose of adding obstacles to this task was two-fold: to stimulate visual, proprioceptive, and skin sensory afferences during locomotion and to encourage the animals to stand higher on their paws. Indeed, during the critical phase of the syndrome, when they are in motion, UVN animals use the positioning of their abdomen on the ground to overcome their instability [28]. 

**Figure 1:**
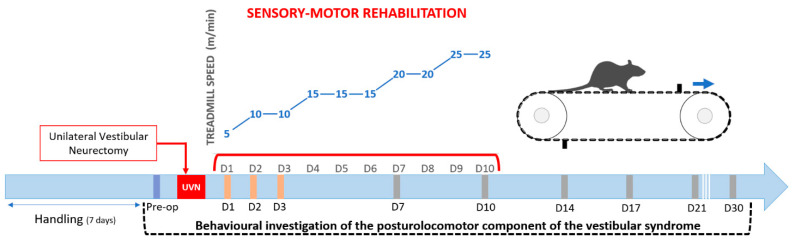
Study design: Details of the procedure used to evaluate quantitatively and qualitatively the vestibular syndrome before and after unilateral vestibular neurectomy. Behavioural investigation of the posturolocomotor component of the vestibular syndrome was made in a first preoperative session (serving as a reference value) and then at 1, 2, 3, 7, 10, 14, 17, 21, and 30 post-lesioned days. The animals in the rehabilitation group were trained from D1 to D10, twice a day on a treadmill task with obstacle clearance. Treadmill speeds were gradually increased as shown in the figure.

To familiarize themselves with the device, the animals were placed on the switched off treadmill for 10 min in a preoperative session. During the first training session, they could again explore the treadmill stationary for 5 min, and then, the treadmill was started at minimum speed (5 m/min). The walking speed of the treadmill was gradually increased (Figure 1) during the rehabilitation protocol. During the critical period, it was set at 5 m/min at D1 and 10 m/min at D2 and D3, which correspond to slow walking speeds. A decrease in the activity of the rodent UVN model was previously observed during the critical period [32], and the aim was to push the animal to perform light activity. The speeds were then adapted according to the rodents’ ability to maintain their position on the treadmill at the previous session. The speed was increased to 15 m/min at D4, 20 m/min at D7, and 25 m/min at D9. These speeds correspond to the average speeds of the first 5 days of training in healthy rats during optimized learning to a running task on a treadmill without obstacles [35]. When the animals stumbled over obstacles but provided an adapted postural response and maintained their walking rhythm, the treadmill speed was maintained. In the event of a fall without immediate resumption of locomotion, the treadmill was stopped, and the speed was gradually increased until the speed for the session was reached. For each session of training, the goal was to have the animals run for a total of 10 min at the correct speed, while minimizing the stress that could be induced by the task.

The vestibular syndrome of the rehabilitated group was assessed in the morning, 30 min after training, on days 1, 2, 3, 7, 10, 14, 17, 21, and 30 post-UVN.

### 2.5. Qualitative Evaluation of the Vestibular Syndrome

The vestibular syndrome induced in the rat is characterized by typical symptoms previously described in studies of the same model (rats UVN model: [28,29,31,32], mice UVN model: [36], cat UVN model: [37]). These classical vestibular symptoms are all present in the acute phase of the syndrome and progressively disappear following vestibular compensation. For this study, we used a cumulative scale previously used by our team [28,29,38,39]. The score assigned to each symptom depends on its severity (tumbling: 5, retropulsion: 4, circling: 3, bobbing: 2, head-tilt: 1).

### 2.6. Quantitative Evaluation of the Vestibular Syndrome

#### 2.6.1. Analysis Device

The second version of the dynamic weight-bearing (DWB2) device (Bioseb, Vitrolles, France) was used to assess the behaviour of our three different experimental groups. This device has been previously described for the assessment of postural imbalance in the same model of vestibulopathy [28,31,34]. It consists of an arena (25 × 25 cm) over which a high-frequency camera is mounted. The floor of the device is covered with a plate of 2000 force sensors. The weight spent by each body part in contact with the ground was assessed automatically in each sensor at a sampling frequency of 30 Hz. The sensors and camera were connected to a computer using the latest DWB2 software version (v2.0.60, Bioseb, Vitrolles, France). The mobility threshold, set at 700 ms, allows the differentiation between static and dynamic behaviour during the analysis. Using the software, the operator then manually identified each paw (front left, front right, rear left, and rear right) and other areas in contact with the floor (tail, abdomen, head, etc.) with the support of the video. 

Each animal was free to explore the arena for 5 min in each session. The pre-operative session was recorded the day before the surgery, and then, the time course of the syndrome was studied every morning following the study design (Figure 1).

#### 2.6.2. Data Analysis

The experimenter can export two types of results: a first file with the average time, area, and weight results of each leg or group of legs and a second file with the detailed results of each press performed on the sensors. The second file can be read by homemade Scilab programmes and allows the extraction of another range of behavioural parameters: postural parameters, circling count, and quantification of support from other areas of the body, such as tail or abdomen, etc. (for more detail about these parameters, see [28]).

The parameters selected for the behavioural assessment of vestibular syndrome are:

- The weight distribution on the left paws (static vs. dynamic), which represents the percentage of weight the animal applies on its left paws. In healthy animals, this percentage is equally distributed between right and left.
- The time spent on the abdomen, in which we add the time of each support located between the four paws of the animal.
- The circling, where each complete and fast lap made by the animal is counted.
- The distance travelled and the average speed of the animal, calculated when the animal is dynamic.

The animal’s barycenter coordinates are calculated at each moment according to the Equation (1):

(1) $$\{\begin{array}{c} \mathrm{Barx}=\frac{\mathrm{FLx}*\mathrm{FLw}+\mathrm{FRx}*\mathrm{FRw}+\mathrm{RLx}*\mathrm{RLw}+\mathrm{RRx}*\mathrm{RRw}}{\mathrm{FLw}+\mathrm{FRw}+\mathrm{RLw}+\mathrm{RRw}}\\ \mathrm{Bary}=\frac{\mathrm{FLy}*\mathrm{FLw}+\mathrm{FRy}*\mathrm{FRw}+\mathrm{RLy}*\mathrm{RLw}+\mathrm{RRy}*\mathrm{RRw}}{\mathrm{FLw}+\mathrm{FRw}+\mathrm{RLw}+\mathrm{RRw}} \end{array}.$$

FLx, FLy, FRx, FRy, RLx, RLy, RRx, and RRy represent the coordinates on the antero-posterior axis and on the medio-lateral axis of the rat of the 4 limbs of the animal (FL: front left paw, FR: front right paw, RL: rear left paw, RR: rear right paw). FLw, FRw, RLw, and RRw represent the weights assigned to each paw of the animal. The barycenter position is calculated for each immobile and 4-legged posture of the animal. This is the rodent equivalent of the center of pressure studied in clinical posturology. Statokinesiograms illustrate the path of the barycenter during an acquisition as well as the position of the 4 paws of the animal at each moment when the barycenter is calculated. The following parameters are related to the movements of the animal’s barycenter:

- The body sway, which is the average of the area of the confidence ellipse at 90% of the points of the barycenter. It is a classical index of postural stability in posturology.
- The amount of energy spent for postural stabilization, which is a ratio between the Speed of displacement of the barycenter and the body sway.

To get rid of inter-individual differences between animals, the posturological results (body sway and amount of energy spent for postural stabilization) and the results relating to distance travelled and average speed were normalized to each animal’s results at the pre-operative session.

### 2.7. Cellular Investigation

#### 2.7.1. Tissue Preparation 

The objective of this procedure is to fix the brain rapidly and uniformly using a 4% intracardiac paraformaldehyde (PFA) perfusion [40]. Rats were deeply anesthetized with a mixture of ketamine 1000 (100 mg/kg) and medetomidine (0.5 mg/kg) after analgesia (buprenorphine 0.05 mg/kg). Intracardiac injection of 300 mL of isotonic saline (0.9% NaCl) was followed by infusion of 300 mL of fresh 4% PFA solution in phosphate buffer ((PB) 0.1 M, pH 7.4). At the end of the perfusion, the brain was extracted from the skull and post-fixed overnight at 4 °C in the same 4% PFA mixture used during the perfusion. Brains were rinsed and cryoprotected by successive transfers into solutions of increasing concentration of sucrose (10%, 20%, then 30% of 0.1 M PB for 72 h at 4 °C). Brains were rapidly frozen with CO_2_ gas and serially cut into 40-µm frontal sections with a cryostat (Leica) for immunochemistry. 

#### 2.7.2. Immunohistochemistry

Immunochemical labelling was performed according to previously validated protocols [9,13,39,41]. Differentiation of the newly generated cells were analysed in UVN and rehabilitation groups that were injected with BrdU on D3 post-UVN and killed on D30. The following primary antibodies were used: mouse anti-BrdU (1:100, Dako, Santa Clara, CA, USA, M0744), rat anti-BrdU (1:100 Abc117-7513), mouse anti-NeuN (1:100, Millipore MAB377, Darmstadt, Germany), rabbit anti-GFAP (1:200, Dako Santa Clara, California, USA, Z0334), rabbit anti-IBA1 (1:2000, Wako 019-19741, Osaka, Japan), and rabbit anti-Olig2 (1:500, Millipore AB9610, Darmstadt, Germany). The following fluorescent secondary antibodies were used: Alexa Fluor 594 goat anti-rat (1:500, Invitrogen A11007, Carslbad, CA, USA), Alexa Fluor 594 donkey anti-goat (1:500, Invitrogen A11005, Carslbad, CA, USA), Alexa Fluor 488 donkey anti-rabbit (1:500, Invitrogen A21206), and Alexa Fluor 488 donkey anti-mouse (1:500, Invitrogen A11029, Carslbad, CA, USA). Brain sections were mounted onto SuperFrost/Plus glass slides (Fischer, Illkirch-Graffenstaden, France) and air-dried before being mounted with Roti^®^Mount FluorCare antifade reagent with the nuclear marker DAPI (Carl Roth, Karlsruhe, Germany).

#### 2.7.3. Cells Count 

For quantification of cells expressing specific markers, 1 in 12 serial sections starting at beginning of VN (relative to bregma, −9.84 mm) to the end of VN (relative to bregma, −13.08 mm), according to the rat brain stereotaxic atlas, were used. The MVN is a multimodal integration nucleus unlike the other vestibular nuclei, which have more distinct functions. It receives multiple afferents (peripheral vestibular receptors, spinal cord, occulomotor nuclei, contralateral vestibular nuclei) and sends efferent to the spinal cord, cerebellum, and thalamus [42], which are essential structures for motor function. For this reason, only sections of the MVN on the lesioned (left) side were evaluated. Quantification of BrdU^+^, NeuN^+^, GFAP^+^, Iba1^+^, and Olig2^+^ cells was counted using confocal imaging with a Zeiss LM 710 NLO laser scanning microscope equipped with a 63×/1.32 NA oil immersion lens. For each marker, immunoreactive positive cells in the MVN were counted using an integrated microscopic counting chamber that delineated the region of interest by a square of 425.10 μm^2^. The average cell counts from the sections were used for statistical analysis.

### 2.8. Statistical Analysis

All statistical analyses were performed using GraphPad Prism software (version 7, GraphPad Software, San Diego, CA, USA). For each of the parameters evaluated, the recorded values are expressed as average ± SEM. To test the effect of UVN and rehabilitation, we performed analysis of variance (two-way ANOVA with repeated measures). For post-hoc analyses, Dunnett’s test were performed to compare values at each post-operative time with pre-operative values and Tukey−Kramer multiple comparison test to compare the results obtained between the three groups and between static and dynamic conditions. The Tukey−Kramer multiple comparison test is adapted for comparison of samples of different size. The results obtained in the three experimental groups are compared with their results obtained in the control condition (pre-operative session). A delay effect is observed when a significant difference is obtained with this condition. Comparisons between the UVN group and the rehabilitation group highlight a rehabilitation effect. The effect of the lesion is observed through the differences obtained between the UVN group and the SHAM group. This last comparison allows us to see possible postoperative effects or effects of habituation to the task that are not visible in the preoperative conditions. The differences between the SHAM and rehabilitation groups are rare and detailed in the results section. Statistical analyses of the cellular data were evaluated by the *t*-test to compare the results of immunohistochemical labelling between the UVN group and the rehabilitation group. *p*-Value < 0.05 was considered as statistically significant (* *p* < 0.05, ** *p* < 0.01, *** *p* < 0.001).

## 3. Results

This study analyses the longitudinal effects of sensory-motor training following a sudden and unilateral suppression of vestibular information on different posturolocomotor and cellular biomarkers. Overall, these results reflect a better recovery of the rehabilitation group compared to the UVN group. They highlight a positive effect of the rehabilitation task developed in this study. From a cellular point of view, the percentage of newly formed cells at D3 and surviving to D30 is higher in the rehabilitation group. These cells mainly differentiate into a microglial phenotype.

### 3.1. Qualitative Evaluation of the Vestibular Syndrome

**Figure 2:**
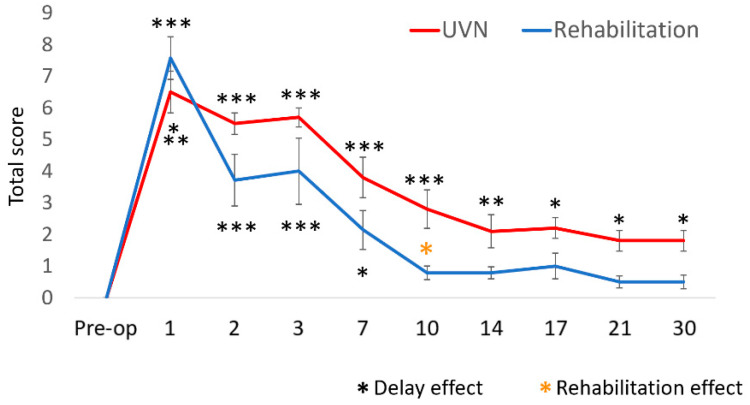
Use of a cumulative qualitative scale for the assessment of vestibular syndrome in the UVN and rehabilitation groups. For both groups, we can observe a critical period where the disorders were at their maximum from D1 to D3 and then a compensated period where the disorders gradually decreased from D7 to D30. The posturolocomotor disorders remained present in the UVN group, while they seemed to disappear in the rehabilitation group.

The results observed in the UVN group show a peak of the disorders from D1 to D3 followed by a progressive decrease of the vestibular disorders. Until D30, the difference with pre-op delay remained significant (*p* < 0.05). For the rehabilitation group, the kinematics of the syndrome is similar from D1 to D3 (*p* < 0.001). At D7, the delay effect was lower in the rehabilitation group (*p* < 0.05. UVN: *p* < 0.001). A significant difference was observed between the rehabilitation group and the UVN group at D10 (*p* < 0.05), and no significant difference was observed with the pre-operative delay from D10 to D30 (Figure 2).

### 3.2. Quantitative Evaluation of the Vestibular Syndrome

#### 3.2.1. Lateral Weight Distribution

**Figure 3:**
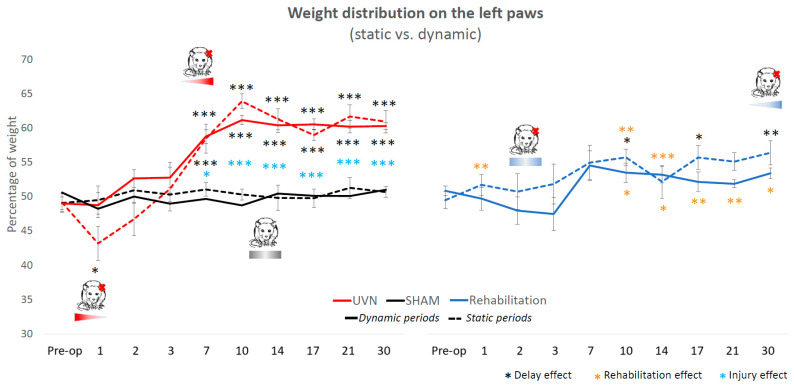
The weight distribution on the lateral axis. This parameter highlights the muscle tone deficits of the three groups of animals. The SHAM group shows a perfectly balanced weight distribution between the right and the left, without any influence of the surgery. In the UVN group, in the critical period, the dynamic weight distribution was well balanced, but there was a shift of the static weight to the right at D1 (*p* < 0.05). At compensated time points, the UVN group applied significantly more weight to the left in both static and dynamic conditions. The weight distribution of the rehabilitated group shows a similar pattern to that of the SHAM group in the dynamic condition. In static condition, there was no deficit in critical period. In the compensated period, the weight shifted to the left at D10, D14, and D30 (delay effect), without significant differences with the other two groups at these times.

The SHAM group had a balanced lateral distribution between right and left during all sessions (Figure 3). This parameter expresses a different kinetics in the UVN group. During the critical period, in dynamic condition, their lateral distribution was balanced: there was no delay effect, and the animal put on average between 50 and 55% of its weight on the left. However, in static condition, there was a significant decrease in the weight applied on the left at D1 (delay effect, *p* < 0.05). In compensated period, from D10 to D30, the UVN animals applied significantly more weight on the left, independently of the static or dynamic character (delay effect: *p* < 0.001 from D10 to D30, lesion effect: *p* < 0.05 at D10 and *p* < 0.001 from D14 to D30). For the rehabilitation group, during all sessions, the weight was distributed between 45% and 55% on the left. The disorders persisted in static condition but not in dynamic: a delay effect appeared for the static condition at D10 (*p* < 0.05), D17 (*p* < 0.05) and D30 (*p* < 0.01). The comparison between the UVN and rehabilitation groups show significant differences at several post-operative delays, in static and dynamic periods. At D1, animals in the rehabilitation group did not shift their weight to the right in static condition (*p* < 0.01). In compensated period, they did not shift their weight to the left in dynamic condition (D10: *p* < 0.05, D14: *p* < 0.05, D17: *p* < 0.01, D21: *p* < 0.01, D30: *p* < 0.01), and in static condition, the lateral distribution of the rehabilitation group was different from the UVN group at D10 (*p* < 0.01) and D14 (*p* < 0.001). 

#### 3.2.2. Distance Travelled

**Figure 4:**
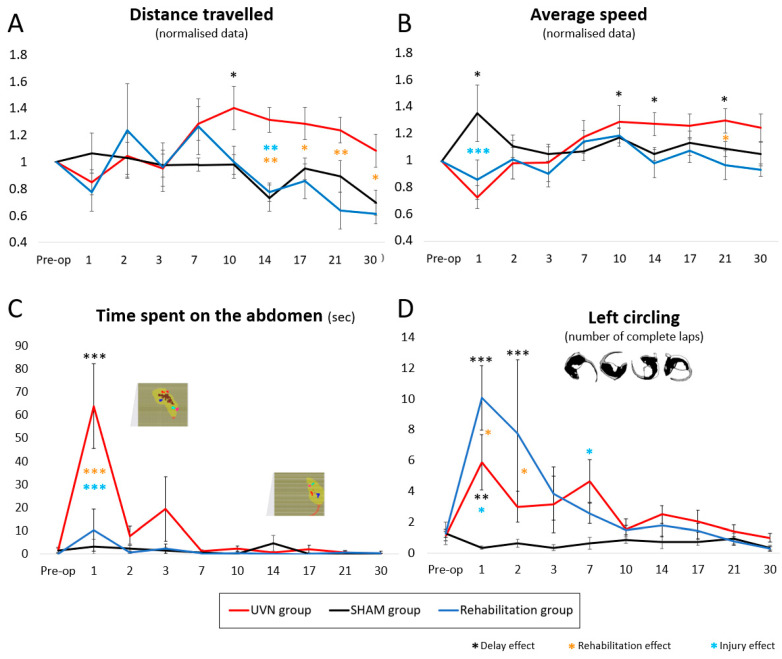
Behavioural activity. This figure summarizes the behavior of the animals when they are in the DWB2. (**A**) Distance travelled. From D10, this parameter increased significantly in the UVN group (delay effect, *p* < 0.05). From D7 to D30, the distance travelled decreased progressively in the SHAM and rehabilitation groups, with significant differences between the rehabilitation group and the UVN group between D14 and D30 (at D14 and D21 *p* < 0.01, at D17 and D30: *p* < 0.05). (**B**) Average speed: the speed increased significantly at D1 for the SHAM group. From D2 to D30, the kinematics of this parameter is similar between the SHAM and rehabilitation groups. In the compensated period, the average speed of the UVN group was higher than that of the other groups (delay effect at D10, D14, and D21 *p* < 0.05, rehabilitation effect at D21 *p* < 0.05). (**C**) Time spent on the abdomen: the UVN group was characterized by the time spent on the abdomen during the critical period (delay effect, rehabilitation effect, and injury effect at D1, *p* < 0.001). The brown pixels in the screenshots represent the sensors activated by a pressure from the abdomen and detected by the DWB2. This behaviour was not observed in the other two groups. (**D**) Left circling: circling was more important in the rehabilitated group at D1 and D2 (delay effect: *p* < 0.001, rehabilitation effect: *p* < 0.05), and then, it decreased and reached lower values than those of the UVN group from D7. In the UVN group, circling behavior was observed from D1 to D7. From D10, the values are similar between the three groups. The animals in the SHAM group did not perform.

From the pre-operative session to D7 post-UVN, no significant difference was observed between the three groups of animals (Figure 4A). At D10, the distance travelled increased significantly for the UVN group (delay effect: *p* < 0.05). This increase in distance travelled was not observed for the SHAM and rehabilitation groups. The SHAM group travelled a significantly shorter distance than the UVN group at D14 (*p* < 0.01). The distance travelled by the rehabilitation group was significantly lower than that of the UVN group from D14 to D30 (D14: *p* < 0.01, D17: *p* < 0.05, D21: *p* < 0.01, D30: *p* < 0.05). 

#### 3.2.3. Average Speed

In the SHAM group, the average speed increased significantly at D1 compared to the pre-operative control delay (*p* < 0.05) (Figure 4B). In the two injured groups (UVN and rehabilitation), the mean speed decreased slightly at D1 and showed significant differences with the SHAM group (rehabilitation group vs. SHAM group: *p* < 0.01; UVN group vs. SHAM group: *p* < 0.001). This parameter then increased slowly for the rehabilitation group and decreased for the SHAM group between D1 and D2 to stabilize at values like those of the preoperative session. In the UVN group, the average speed gradually increased from D1 to D10, then remained stable from D10 to D30. A delay effect was observed in this group at D10, D14, and D21 (*p* < 0.05). At D21, the mean velocity was significantly lower in the rehabilitation group compared to the UVN group (*p* < 0.05).

#### 3.2.4. Time Spent in the Abdomen

The SHAM and rehabilitation groups did not, or very little, place their abdomen on the sensors from the preoperative session to D30 post-surgery. At D1, the UVN group spent significantly more time with the abdomen placed on the floor compared to the preoperative session (*p* < 0.001) and compared to the rehabilitation (*p* < 0.001) and SHAM (*p* < 0.001) groups. At D2 and D3, the UVN group continued to spend time with abdomen on the floor, without significant difference with the other groups and without delay effect; then, from D7 to D30, the pose time became negligible (Figure 4C). 

#### 3.2.5. Left Circling

Before surgery, none of the three groups performed left hand circling (Figure 4D). The number of fast laps per session ranged from 1.29 ± 0.72 laps for the SHAM group to 1.29 ± 0.26 laps for the rehabilitation group. The SHAM group did not perform circling after surgery: the number of turns per acquisition varied very little from the preoperative session to the D30 delay. At D1 and D2, the rehabilitation group performed significantly more circling than the SHAM (*p* < 0.001) and UVN (*p* < 0.05) groups and had a delay effect (*p* < 0.001). Then the number of rotations per acquisition performed by this group progressively decreased at D3 and D7, where it became lower than that of the UVN group. From D10 to D30, the values obtained are similar to those of the SHAM group. The kinematics of this parameter is different for the UVN group. The animals of the UVN group circling from D1 to D7, with a delay effect present at D1 (*p* < 0.01) and a significant difference with the SHAM group at D7 (*p* < 0.05). It then re-joined the values of the SHAM groups and was rehabilitated between D10 and D30.

#### 3.2.6. Posturological Parameters

**Figure 5:**
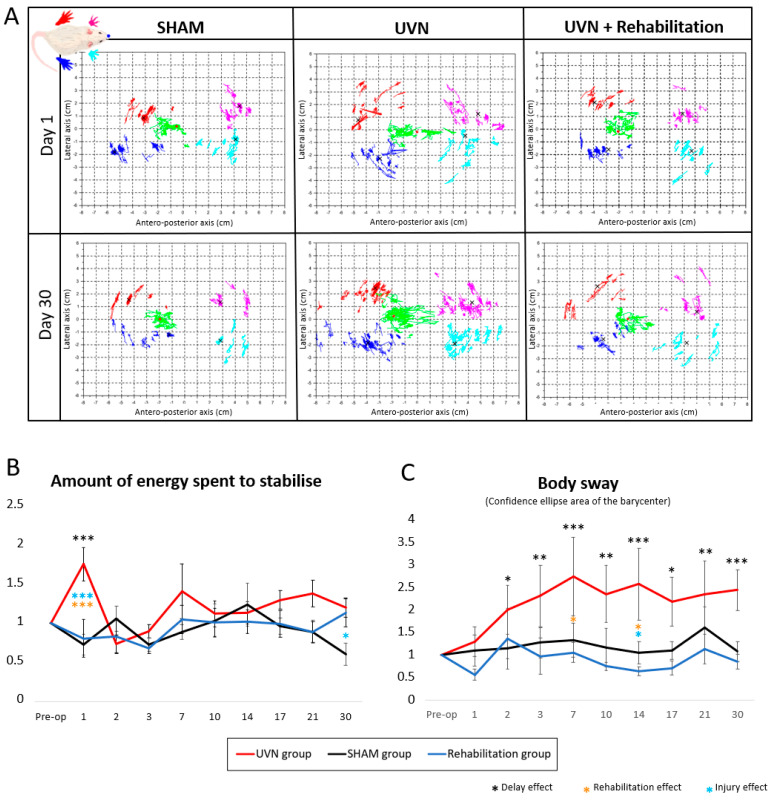
Posturographic parameters. (**A**) Rodent statokinesiograms: representation of the kinetics of the positions of the animals’ paws and the barycenter. The green clusters correspond to the positions of the barycentre during an acquisition, the red, blue, magenta and cyan dot clouds correspond to the left and right hind paws, and the left and right forepaws respectively. The different columns indicate the group of the animal from which the plot is extracted, and the lines indicate the acquisition time (day 1 and day 30 post-op). This figure is illustrative and allows the visualisation of the postural imbalance of the different groups in the study. These are examples of statokinesiograms obtained for one rat in each group. At day 1, the plot of the rehabilitation group showed less instability than the UVN group but more than the SHAM group. At D30, the statokinesiograms of the SHAM and rehabilitation groups seemed identical, whereas the instability seemed to persist in the UVN group. The following parameters quantify postural instability in the rodent. (**B**) Amount of energy spent to stabilize. From the preoperative session to post-injury day 21, the evolution of this parameter is similar for the rehabilitation group and the SHAM group. At D1, the UVN group spent significantly more energy than the other groups (*p* < 0.001) and showed a delay effect (*p* < 0.001). At D30, the SHAM group expended significantly less energy to stabilize than the UVN groups. (**C**) The body sway is a stability index frequently used in clinical posturology. This parameter highlights an instability that progressively sets in for the UVN group and that lasts (delay effect from D2 to D30: 0.05 < *p* < 0.001). The SHAM and rehabilitation groups managed to maintain their postural stability at all postoperative time points.

Figure 5A shows the statokinesiograms of animals present in the three groups at D1, which is the peak of expression of vestibular symptoms following UVN and at D30, which is the latest compensated delay. The clusters of red, pink, cyan, and navy-blue dots represent the successive positions of the four paws of the animals (respectively: rear left, front left, rear right, and front right) at each period of an acquisition where the animal is motionless and on all four paws. Each black cross represents the average of a cloud. The cloud of green dots represents the successive positions of the barycenter calculated at each moment, always when the animal is motionless on its four paws, and the red dot represents the average position of the barycenter during an acquisition. These plots highlight a greater dispersion of the different dot clusters in the UVN group, in critical and compensated periods. The rehabilitation group also seemed to present more scattered clouds at D1, but the statokinesiogram extracted from the rehabilitation group at D30 is closer to that obtained from the SHAM group than from the UVN group.

This figure is illustrative. The body sway and the amount of energy spent to stabilize allow a quantitative analysis of the dispersion of the barycenter when the animals were immobile (Figure 5B,C).

The body sway quantifies the dispersion of the barycenter for each period when the animal is motionless and on all four legs. It is an index of instability commonly quantified in clinical posturology. In SHAM and rehabilitation groups, the normalized data were close to 1 in all acquisition sessions. In the UVN group, the body sway progressively increased from D1 to D2. From D2, a delay effect set in until D30 (D2: *p* < 0.05, D3: *p* < 0.01, D7: *p* < 0.001, D10: *p* < 0.01, D14: *p* < 0.001, D17 *p* < 0.05, D21: *p* < 0.01, D30: *p* < 0.0). The mean body sway of the UVN group was significantly higher than that of the rehabilitation group at time points D7 (*p* < 0.05) and D14 (*p* < 0.05). At D14, it was also significantly higher than the values obtained in the SHAM group (*p* < 0.05).

The amount of energy spent to stabilize is calculated from a ratio between the speed of the barycenter and the body sway. This is an estimate of the effort expended to increase its stability. In the UVN group, this parameter increases significantly between the preoperative session and D1 (*p* < 0.001). At D1, the values obtained were also significantly higher than the SHAM (*p* < 0.001) and rehabilitation (*p* < 0.001) groups. From D2 to D21, no difference was observed between the three groups. At D30, the UVN and rehabilitation groups expended significantly more energy to stabilize than the SHAM group (UVN vs. SHAM group: *p* < 0.05, rehabilitation vs. SHAM group: *p* < 0.05).

### 3.3. Influence of the Rehabilitation Protocol on Cell Populations of the Ipsilateral Vestibular Nucleus

**Figure 6:**
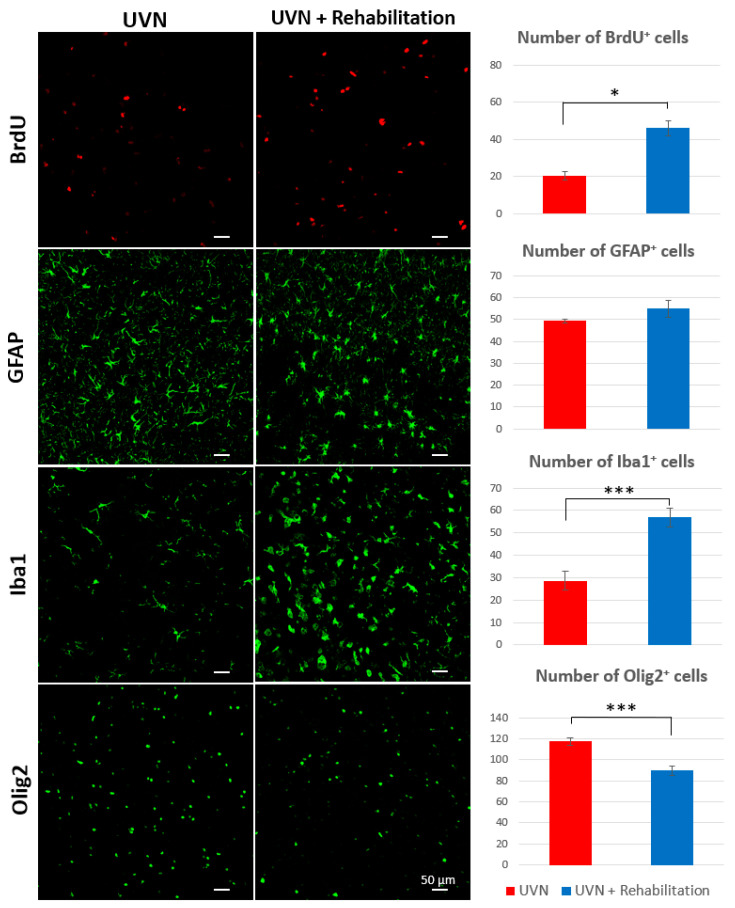
Cell population counts in the medial vestibular nucleus of rats in the UVN and rehabilitation groups 30 days after injury. BrdU injection 3 days after injury was used to analyse cell survival in the compensated phase of the vestibular syndrome. On the left are images of immunohistochemical staining taken with a confocal microscope. The newly generated cells (BrdU^+^) are marked in red. Green labelling highlights astrocytes (GFAP^+^), microglia (Iba1^+^), and oligodendrocytes (Olig2^+^). On the right side of the figure are histograms showing the number of immunoreactive BrdU, GFAP, Iba1, and Olig2 cells. Compared to the UVN group, the rehabilitation group had significantly more BrdU^+^ cells (*p* < 0.05), more microglial cells (*p* < 0.001), and fewer oligodendrocytes (*p* < 0.001).

**Figure 7:**
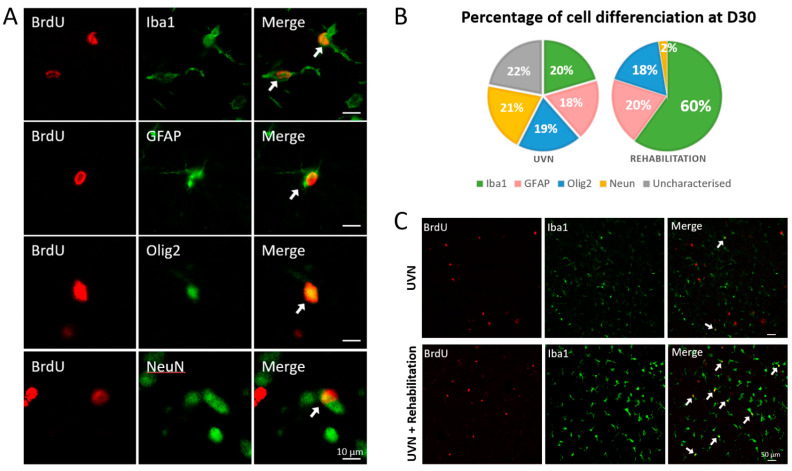
Differentiation of newly generated cells in the deafferented MVN of rats in the UVN and rehabilitation groups 30 days after the injury. BrdU is used as a cell proliferation marker. It is injected 3 days after UVN, and the cell quantification was made on the same rats sacrificed 30 days after UVN. (**A**) Immunostaining images taken with a confocal microscope. BrdU nuclei are marked in red, and cell phenotype markers (Iba1, GFAP, Olig2, and NeuN) are green. (**B**) Pie charts illustrating the percentage of GFAP^+^, IBA1^+^, Olig2^+^, and NeuN^+^ cells among the BrdU^+^ cells in the UVN and rehabilitation groups. The percentages are globally balanced in the UVN group (about 20% for each cell type and 20% of uncharacterised cells). The rehabilitation group favours a microglial differentiation phenotype (60%), and the proportion of newly formed neurons is very low (2%). (**C**) double immunostaining images taken with a confocal microscope in the deafferented MVN of the UVN group (upper part) and the rehabilitation group (lower part). The BrdU^+^ nuclei are shown in red, and the Iba1^+^ cells are shown in green. We show here that the rehabilitation protocol increases the microgliogenesis compared to the UVN untrained group (see white arrows).

Cellular differentiation phenotype of VN after UVN has been described in detail in the literature [9,10,11,33]. A peak of neurogenesis was observed at D3 in the rat UVN model, concomitant with a significant increase in the number of astrocytes, microglial cells, and oligodendrocytes. When the animal has compensated for most of its disorders, the expression of these different cell types decreases but remains significantly higher than preoperative values. Newly formed cells at D3 differentiate equitably between these different cell types at D30 (see [9] for more details). In this study, we were interested in the influence of our rehabilitation protocol, 30 days after injury, on the different cell types present in the ipsilateral VN (Figure 6) as well as on the cellular differentiation of newly formed cells at D3 (Figure 7).

#### 3.3.1. Cell Population Counts

The number of BrdU^+^ cells at D30 was doubled in the rehabilitation group (46.1 ± 4.05) compared to the UVN group (20.4 ± 2.27, *p* < 0.05). Similar results were observed for Iba1^+^ cells, which were much higher in the rehabilitation group than in the UVN group (*p* < 0.01). On the contrary, the number of Olig2^+^ cells was significantly lower in the rehabilitation group (*p* < 0.01). We can note that it is equivalent to the number of Olig2^+^ cells in preoperative session obtained in a previous study of our team [9]. The results obtained for GFAP labelling are similar between the two groups, and no statistical difference is observed.

#### 3.3.2. New Cells Differentiation

The differentiation of the newly generated cells was analysed in the UVN and rehabilitation groups. These animals were injected with BrdU at D3 post-UVN and sacrificed after behavioural evaluation of the syndrome at D30. The results obtained in the UVN groups indicate that the new cells generated by the lesion were equitably distributed between the different cell types (Iba1: 20%, GFAP: 18%, Olig2: 19%, Neun: 21%). A total of 22% of the BrdU^+^ cells have an uncharacterized phenotype. These proportions differed in the rehabilitation group. Indeed, 60% of the BrdU^+^ cells differentiated into microglia and only 2% into neurons for the rehabilitation group. The results are similar between the two groups for GFAP and Olig2 markers. In addition, there was no uncharacterized phenotype in the rehabilitation group.

## 4. Discussion

This study highlights the beneficial effect of early and regular sensory-motor training on a rodent model of APV. The aim was to increase motor and sensory inputs (tactile and proprioceptive) in the VN during all stages of vestibular compensation. We used a complex motor task: progressive training on a treadmill with obstacles clearance. We followed the recommendations applied in clinical practice to optimize the beneficial effects of a vestibular rehabilitation protocol [30]. The consequences of this sensorimotor rehabilitation were evaluated from a behavioural and cellular approach. We show that vestibular rehabilitation induces a faster recovery of posturolocomotor deficits during vestibular compensation associated with a decrease in neurogenesis and an increase in microgliogenesis in the deafferented medial vestibular nucleus. This study reveals for the first time a part of the underlying adaptive neuroplasticity mechanisms of vestibular rehabilitation.

### 4.1. Recovery Kinetics of Postural and Locomotor Functions 

The results of the behavioural evaluations show a rapid restoration of balance and a decrease in posturolocomotor disorders in the rehabilitation group compared to the UVN group. This better recovery is observed on the qualitative analysis of the vestibular components of the syndrome, where a return to preoperative values was observed as early as D10, while the untrained UVN group maintained signs of vestibular disorders throughout the compensated period of the syndrome. Quantitative analysis of the vestibular syndrome allows us to evaluate more precisely the kinetics of static and dynamic vestibular disorders. Behavioural parameters recorded under static condition include times when the animal stops voluntarily in the device, without any constraint. This method of analysis is therefore carried out under ecological conditions. From the different parameters extracted, we highlighted different recovery kinetics between the acute and compensated phase of the syndrome.

#### 4.1.1. Immediate Restoration of Static Deficits 

The results obtained in the static condition have one thing in common: the rehabilitation group showed fewer static disorders than the UVN group from D1 post-UVN. Indeed, the quantification of the static weight distribution on the lateral axis in the UVN group was characterized by a shift of the weight to the right side (intact side) at D1, reflecting a muscular hypotonia on the left side (ipsilesional side) due to the hypoactivation of the ipsilesional vestibulospinal pathways, then a second shift of the overall weight to the left from D7 to D30, reflecting the implementation of a new postural strategy [28,31,34]. The recovery kinetics of this parameter is completely different in the rehabilitation group. In this group, the weight distribution in the lateral axis is very close to what is observed in the sham group, which attests to the beneficial contribution of rehabilitation to postural stability. The rare support on the abdomen of the rehabilitation group underline the faster recovery of the antigravity muscle tone of the rehabilitation group. A recent study found evidence of a remodelling of somatosensory cortical maps of the hind paws the day after the lesion in the same APV model as early as one hour after the surgery [34]. According to this study, the application of more weight on the ipsilesional paws is a compensatory strategy implemented to overcome these changes in cortical networks. The rehabilitation task used here allows, among other things, to stimulate cutaneous and proprioceptive afferents. The increase of cutaneous sensory inputs in the VN could act on the remodelling of cortical maps from the first training session. The stimulation of the vestibulo-thalamo-cortical pathways could then help to maintain static balance from the critical period. The posturological parameters also reflect the rapid functional restoration of static behavioural parameters. Indeed, we have shown in a previous study [28] that UVN animals spend much more energy to stabilize their posture at the start of the acute period and then decrease their energetic effort to stabilize themselves, which led to an increase of a chronic static instability in this group. The results obtained in the rehabilitation group are like those of the SHAM group and remained relatively stable during all the acquisition sessions, from the first day after vestibular injury. Data from the literature on vestibular compensation agree that the recovery kinetics of static deficits are faster than dynamic deficits. The restoration of static deficits is an intrinsic process dependent on the return to electrophysiological equilibrium between the intact and the deafferented VN [43]. The increase of sensory inputs in the VN could accelerate early plasticity mechanisms in the VN [9,10,11,12,13] and thus allow a faster compensation of static deficits. 

Nevertheless, animals in the rehabilitation group tended to apply more weight on the ipsilesional side at D17 and D30 compared to the preoperative condition, and the energy spent to stabilize was similar to that of the UVN group and significantly different from that of the SHAM group at D30. These results indicate a deficit in the maintenance of static balance that appears one week after the end of the rehabilitation protocol. It would be interesting to observe the effects of additional training sessions after D10 to see whether these static deficits observed at late post-lesional delays reflect a lack of regular sensory influxes in the VN.

#### 4.1.2. Later Dynamic Recovery

The rehabilitation task developed in this study is a complex motor task that requires multimodal integration. This task stimulates the motor pathways as well as the visual and proprioceptive sensory pathways, which all pass through the VN, considered as a first integrating relay of this heteromodalitary information. A balance between the vestibular reflex loops involving these different motor and sensory modalities is necessary to maintain dynamic balance [44]. In the UVN group, dynamic deficits are maintained over the long term. An increase in animal activity was observed, which has already been reported in the same model during exploration of a classical open field [32]. Dynamic deficits following vestibular pathology have also been observed in human clinical practice and are thought to reflect central vestibular pathologies [45]. Persistent dynamic deficits in the UVN group could result from an insufficient reorganization of the central compensatory networks. It is known that regular physical and complex training can improve postural balance, even in the face of a disturbance of the vestibular system [46]. In this study, the results obtained in the rehabilitation group show a better recovery of long-term dynamic deficits. The increase in sensorimotor influxes induced by the rehabilitation task allows to optimize the central compensatory mechanisms. Nevertheless, unlike static deficits, animals in the rehabilitation group show dynamic deficits during the acute period of the syndrome. The distance travelled and average speed of the animals in the rehabilitation group was like that of the UVN group up to D7, and circling was even accentuated in the rehabilitation group at D1 and D2. The VN project to the striatum through the parafascicular nucleus of the thalamus and circling is a behavioural consequence of an imbalance in this vestibulo-thalamo-striatal network [47]. This behaviour has been observed in several animal models of vestibular pathology [29,31,32,36,48]. Besides, the striatum integrates multisensory information and works in collaboration with the motor cortex using cortico-striatal loops for movement planning and execution. In animals subjected to rehabilitation, the additional sensorimotor inputs induced by the task converge on the VN but also on the striatum, for which the vestibular lesion also caused an imbalance. Potentiating sensory afferents in the acute phase in an already unbalanced striatum would probably accentuate the circling phenotype. However, the circling disappears more quickly in the rehabilitation group due probably to a more rapid return to equilibrium of this vestibulo-thalamo-striatal network.

In this study, the investigation of the dynamic behavior shows that training does not have an immediate effect, but it does allow faster and more efficient dynamic compensation. The restoration of dynamic deficits results both from the electrophysiological homeostasis in the VN but also from extrinsic mechanisms calling on other sensory and motor pathways; one speaks of sensory substitution [8,49]. The increase in sensorimotor stimuli applied from the acute phase of the syndrome significantly accelerates the restoration of dynamic equilibration parameters from the third day post-UVN by maintaining them for the long term.

### 4.2. Neurobiological Correlates of Sensorimotor Rehabilitation in the VN

We bring in this work the first demonstration of plasticity mechanisms reactive to rehabilitation, which are expressed in the deafferent MVN and which could underlie the best functional recovery observed in rehabilitated animals. We show in this study that a protocol of progressive sensorimotor rehabilitation carried out from the critical period of the syndrome until its compensation leads to an increase in cell survival in the deafferented MVN of the rehabilitation group (Figure 6). These results show that rehabilitation has an impact on the survival rate of newly generated cells in the deafferented MVN. In other brain structures, like the hippocampus, it has been known for a long time that running increases cell proliferation [50,51] Data from the literature also show that training increases the synthesis of BDNF, which is a factor that promotes cell survival and proliferation [52,53,54]. We have shown in the UVN feline model that treatment with BDNF considerably increases the level of cell proliferation and survival in all deafferented VN [11]. In view of these cellular observations, it is quite possible that our rehabilitation protocol increases BDNF synthesis and release in the deafferented vestibular environment, making it favourable to the expression of the various steps of neurogenesis (proliferation, survival, and differentiation). 

We also observed in this work a strong impact of the rehabilitation on the glial reaction. In the UVN rehabilitation group, the microglial reaction was significantly increased in the deafferented MVN; conversely, the number of oligodendrocytes was significantly reduced, while the astrocytic reaction remained constant compared to the non-rehabilitated UVN group. In addition, we show that most cells generated three days after UVN prioritize microglial differentiation to the detriment of neurogenesis in deafferented MVN (Figure 7). Due to the low percentage of new neurons in the rehabilitation group, the production of more myelin is not necessary. This explains the decrease in oligodendrocyte numbers observed in the rehabilitation group. The microglial phenotype appears to be the primary target of rehabilitation, and several arguments can support this finding.

Several studies report an increase in the microglial reaction after vestibular deafferentation. A significant increase in the number of microglial cells was observed three days after UVN persisting up to 30 days post-lesion [9,10,11]. Other groups report the same observation: strong microglial reactions were also observed in the rat deafferented VNs after unilateral labyrinthectomy (UL) [55,56]. The microglial reaction would promote the plasticity mechanisms in the deafferented vestibular environment and, in particular, the level of excitability, which is crucial for functional recovery. We recently demonstrated a down regulation of the excitability marker, such as the cation-chloride co-transporter KCC2, during the first three days after UVN [11]. This remodelling favours a depolarizing action of GABA, which could facilitates the VN electrophysiological homeostasis, a key parameter leading to a faster functional recovery. Numerous studies have shown that microglia could be at the origin of this mechanism by releasing BDNF, which in turn activates TrkB receptors and downregulates KCC2 expression [57,58,59]. This beneficial action of the microglia on the return of excitability to a physiological level would then be sufficiently effective to spare the deafferented vestibular environment the use of the neurogenesis process.

Moreover, BDNF administration improves vestibular compensation, leads to an increase in dendritic regrowth in the ipsilateral VN, and is accompanied by an increase in microglial differentiation [11]. Release of BDNF play a neuroprotective role that may explain the low number of newly generated neurons in the deafferented MVN after UVN. The newly created microglial cells would have protected neurons already present in the VN while promoting synaptic connections and dendritic regrowth. Indeed, microglial cells can participate in modulating the synaptic strength of sensory afferents converging to VN to promote sensory substitution mechanisms during compensation. Previous studies in UL macaques have shown increased sensitivity to proprioceptive inputs of neurons in the VN [60,61]. Proprioceptive synapses, which in non-pathological conditions are silent, become activated after the lesion. Proprioceptive signals are thus up-weighted during vestibular compensation probably due to a remodelling of synaptic strength. It has been shown that activation of microglia is sufficient to rapidly induce synaptic modulation and synaptic facilitation [62]. The increase in sensory inputs induced by the rehabilitation task then explains the increase in the number of microglial cells in the deafferented VN.

The increased microgliogenesis observed after rehabilitation could act on inflammatory mechanisms by promoting the neuroprotective action of microglia. To our knowledge, no further studies have been performed on microglial inflammatory phenotypes following UVN. A more in-depth study could lead to a new drug approach.

### 4.3. Clinical Relevance

In the field of vestibular rehabilitation, research is progressing mainly using protocols conducted in clinics on vestibular patients [21,22]. The clinical practice of vestibular rehabilitation protocols aims to improve and accelerate the central compensation of the patient. This study shows the potential effects of rehabilitation on acute unilateral vestibular hypofunction in animals that can be compared to those observed in humans [63]. A recent meta-analysis demonstrates a significant effect of reduced vertigo frequency in rehabilitated patients compared to groups without intervention [21]. The training protocol here seeks to improve postural as well as locomotor stability. Unlike many protocols used in clinical practice, little involvement of gaze stabilization in space is induced although the existence of obstacles to overcome may contribute. Clinical practice remains complex, however, and subject to compensatory and substitution processes, including, for example, compensation of the vestibulo-oculomotor reflex and substitution by the saccadic system that occur early on among adaptive strategies [64]. The task used here is more global and requires the concomitant action of several sensory systems, and our results show that it is beneficial for postural and locomotor symptoms in this APV rodent model. Once again, it is shown that post-APV rehabilitation should be very early and that it is probably not necessary to worry about the fact that the results obtained may vary from one individual to another during the early post-critical period, which is often observed in patients. This observation could bring a new look on protocols in clinical practice. The physiotherapist can find here an argument in favour of regular and personalised care. However, several points of interest, possibly determining, concerning the rehabilitation protocol and easily adaptable to the patients can be noted: multi-daily sessions, adaptation to the subject’s capacities during the whole treatment, systematic research of the performance during each session, and ecological task.

## 5. Conclusions

In this study, we have developed a sensory-motor rehabilitation protocol adapted to the posturolocomotor deficits of the rat UVN model. This protocol has shown its efficiency on the recovery kinetics of vestibular syndrome by accelerating and improving its posturolocomotor components. We demonstrate for the first time an increase in microgliogenesis in the deafferented MVN after a sensory-motor rehabilitation protocol that underlies the establishment of additional adaptive plasticity mechanisms following a sensory-motor rehabilitation task, which is done to the detriment of neurogenesis. The study of vestibular rehabilitation in animal models will allow a better understanding of the cellular mechanisms that promote the functional recovery during vestibular disorders. A better knowledge and understanding of microglia mechanisms of action in the VN could provide new pharmaceutical targets for a better management of vestibular pathologies.

## Data Availability

The data presented in this study are available on request from the corresponding author.
